# Are shared decision making studies well enough described to be replicated? Secondary analysis of a Cochrane systematic review

**DOI:** 10.1371/journal.pone.0265401

**Published:** 2022-03-16

**Authors:** Titilayo Tatiana Agbadjé, Paula Riganti, Évèhouénou Lionel Adisso, Rhéda Adekpedjou, Alexandrine Boucher, Andressa Teoli Nunciaroni, Juan Victor Ariel Franco, Maria Victoria Ruiz Yanzi, France Légaré

**Affiliations:** 1 Centre de recherche sur les soins et les services de première ligne de l’Université Laval (CERSSPL-UL), Quebec, Canada; 2 Tier 1 Canada Research Chair in Shared Decision Making and Knowledge Translation, Université Laval, Quebec, Québec, Canada; 3 Family and Community Medicine Division, Hospital Italiano, Buenos Aires, Argentina; 4 Research Department, Instituto Universitario Hospital Italiano, Buenos Aires, Argentina; 5 Alfredo Pinto Nursing School, Federal University of the State of Rio de Janeiro - UNIRIO, Rio de Janeiro, Brazil; 6 Department of Family Medicine and Emergency Medicine, Faculty of Medicine, Université Laval, Quebec, Canada; Duquesne University, UNITED STATES

## Abstract

**Background:**

Interventions to change health professionals’ behaviour are often difficult to replicate. Incomplete reporting is a key reason and a source of waste in health research. We aimed to assess the reporting of shared decision making (SDM) interventions.

**Methods:**

We extracted data from a 2017 Cochrane systematic review whose aim was to determine the effectiveness of interventions to increase the use of SDM by healthcare professionals. In a secondary analysis, we used the 12 items of the Template for Intervention Description and Replication (TIDieR) checklist to analyze quantitative data. We used a conceptual framework for implementation fidelity to analyze qualitative data, which added details to various TIDieR items (e.g. under “what materials?” we also reported on ease of access to materials). We used SAS 9.4 for all analyses.

**Results:**

Of the 87 studies included in the 2017 Cochrane review, 83 were randomized trials, three were non-randomized trials, and one was a controlled before-and-after study. Items most completely reported were: “brief name” (87/87, 100%), “why” (rationale) (86/87, 99%), and “what” (procedures) (81/87, 93%). The least completely reported items (under 50%) were “materials” (29/87, 33%), “who” (23/87, 26%), and “when and how much” (18/87, 21%), as well as the conditional items: “tailoring” (8/87, 9%), “modifications” (3/87, 4%), and “how well (actual)” (i.e. delivered as planned?) (3/87, 3%). Interventions targeting patients were better reported than those targeting health professionals or both patients and health professionals, e.g. 84% of patient-targeted intervention studies reported “How”, (delivery modes), vs. 67% for those targeting health professionals and 32% for those targeting both. We also reported qualitative analyses for most items. Overall reporting of items for all interventions was 41.5%.

**Conclusions:**

Reporting on all groups or components of SDM interventions was incomplete in most SDM studies published up to 2017. Our results provide guidance for authors on what elements need better reporting to improve the replicability of their SDM interventions.

## Background

Shared decision making (SDM) is a patient-centered approach whereby clinicians and patients work together to make decisions based on the best available evidence and according to the patient’s values and preferences [1, 2]. SDM is particularly relevant when there are different preference-sensitive options to consider [3]. It has been shown that SDM has positive effects on patients’ health outcomes and their satisfaction with the decisions made [4, 5]. It improves the experience of both patients and health professionals [6, 7]. Indeed, SDM represents a set of essential actions (e.g. define/explain problem, present options, discuss pros/cons) [8] that must be achieved by patients and health professionals together [9]. It is possible to specify behaviors that each party must adopt for SDM to occur and then to intervene to increase adoption of these behaviors [10, 11].

Despite its benefits, SDM has not yet been widely adopted in clinical practice [12]. Many barriers to SDM have been identified in the literature [13, 14]. Some studies demonstrate missed opportunities for SDM in various contexts [15, 16]. For example, in one study oncologists rarely expressed that a treatment decision needed to be made in consultations concerning cancer care [15]. Another study in mental health contexts suggested that patients could participate more in goal setting [16].

Numerous interventions using behavioral change strategies have been developed to increase the adoption of SDM by health care professionals and the involvement of patients in their own care [13]. A Cochrane systematic review informed us on interventions for increasing the use of SDM by health professionals in a variety of contexts [17]. Many interventions targeting patients, healthcare professionals or both were multi-component interventions designed to change health professionals’ performance and behavior, such as the way they present information and interact with patients [17]. Authors concluded that the certainty of the evidence was too low to determine if the interventions were effective or not for increasing the use of SDM, and explained that this was mainly due to poor reporting of results [17].

The completeness of reporting of interventions in primary studies is one of the key challenges for assessing their quality [18]. Incomplete reporting limits our ability to learn through systematic reviews, meta-analyses, and other forms of research synthesis [19]. It also makes it difficult to replicate or scale up effective interventions or learn from ineffective ones [20]. Incomplete reporting of intervention components makes it hard to identify the active ingredients linked to improvement [21]. In sum, incomplete reporting is a major source of waste in health research [22].

Initiatives such as the EQUATOR (Enhancing the QUAlity and Transparency Of health Research) Network aim to improve the reliability and value of published health research literature by promoting transparent and accurate reporting and wider use of robust reporting guidelines [23]. The template for intervention description and replication (TIDieR), a reporting guideline, provides a structured framework for reporting interventions [22].

Reporting quality was not covered by the parent Cochrane review. Therefore, in order to promote better replication and scaling up of effective SDM interventions, we aimed to describe the quality of reporting of SDM interventions included in the review.

## Methods

We performed a secondary analysis of a 2017 Cochrane systematic review entitled “Interventions for increasing the use of shared decision making by healthcare professionals” [17]). The Cochrane review sought eligible studies in CENTRAL, MEDLINE, Embase, Health Technology Assessment Database, NHS Economic Evaluation Database, PubMed, CINAHL EBSCO, PsycINFO Ovid, two clinical trial registries and proceedings of relevant conferences. The 87 studies included in the 2017 Cochrane review reported on interventions targeting either patients, health professionals or both [17]. The present study, a secondary analysis of the Cochrane review, analyzed all 87 included articles. Details on information sources, search methods, study selection, data collection and analysis, assessment of risk of bias, data synthesis and summary of findings of the original systematic review are available in full in the Cochrane Library [17].

There are currently no reporting guidelines specifically for secondary analyses of systematic reviews. Thus we used the Preferred Reporting Items for Systematic reviews and Meta-Analyses (PRISMA) checklist [24] (S1 Checklist) and the Guidelines for reporting meta-epidemiological methodology research [25] to report this study.

### Data collection

#### Theoretical underpinnings

This secondary analysis used the Template for Intervention Description and Replication (TIDieR) checklist [22] as a reference for extracting items reported in SDM interventions. The TIDieR checklist was inspired by the SPIRIT checklist (Standard Protocol Items: Recommendations for Interventional Trials) and the CONSORT statement (Consolidated Standards of Reporting Trials) created to address poor reporting in protocols and randomized control trials [19, 20, 22]. While CONSORT and SPIRIT cover some elements of reporting on interventions (e.g. outcomes, population), TIDieR provides a complementary list of items to offer guidance for more complete reporting with the ultimate purpose of improving the efficacy and replicability of interventions [22].

The TIDieR checklist contains 12 items, or questions: 1) “Brief name?” i.e. a name or a phrase that describes the intervention; 2) “Why?” i.e. any rationale, theory, or goal of the elements essential to the intervention; 3) “What (materials)?” i.e. any physical or informational materials used in the intervention, including those provided to participants or used in intervention delivery or in the training of intervention providers; 4) “What (procedures)?” i.e. procedures, activities, and/or processes used in the intervention, including any enabling or support activities; 5) “Who?” i.e. expertise, background and any specific training given for each category of intervention provider (e.g. psychologist, nursing assistant); 6) “How?” i.e. the modes of delivery (such as face-to-face, internet or telephone) of the intervention and whether it was provided individually or in a group; 7) “Where?” i.e. the type(s) of location(s) where the intervention occurred, including any necessary infrastructure or relevant features; 8) “When and how much?” i.e. the number of times the intervention was delivered and over what period of time, including the number of sessions, the schedule, and the duration, intensity or dose; 9) “Tailoring?” i.e. if the intervention was planned to be personalized, titrated or adapted, what, why, when, and how; 10) “Modifications?” i.e. if the intervention was modified during the course of the study, what changes were made (what, why, when, and how); 11) “How well (planned)?” i.e. if intervention adherence or fidelity was assessed, how and by whom, and if any strategies were used to maintain or improve fidelity; 12) “How well (actual)?” i.e if the intervention adherence or fidelity was assessed, the extent to which the intervention was delivered as planned [22]. Unlike items 1 to 8, items 9 to 12 are conditional, i.e. reporting is only required if the intervention was planned to be tailored (item 9), if it was modified during the course of the study (item 10), or if adherence or fidelity were assessed (items 11 and 12) (See Table 1) [22].

**Table 1:** TIDieR items and examples of data extracted.

TIDieR item number	TIDieR item name	Data extracted	Examples
**1**	**Brief name**	a) Name	MyAsthma, *Fiks 2015*
		b) Acronym	BRIDGES (Building Recovery of Individual Dreams and Goals). *Pickett 2012*
		c) Brief description of the intervention	Behavioral SDM intervention for inpatients with schizophrenia. *Hamann 2017*
**2**	**Why**	a) Goal	“The CCPP package for patients aims to change patient behavior, and through these changes, alter physician behavior.” *Butow 2004*
		b) Rationale	“Decision-making preferences of patients with cancer are not always met, and often oncologists do not elicit these. Oncologists’ perceptions may be inconsistent with patients’ stated preferences, for example, in elderly patients [19]. These difficulties are heightened when discussing a clinical trial [20–23]. These issues suggest that targeted training in SDM is warranted. Communication training in decision making regarding standard care has been shown to be effective in a randomised controlled trial in the family practice setting.” *Bernhard 2011*
		c) Theory of the intervention	“Developed in 1986 after an extensive literature review and needs assessment, it was built around a new model of clinician-patient communication, the “4E Model” (Engage, Empathize, Educate, and Enlist) (Keller and Carroll, 1994), which includes key clinician-patient communication competencies detailed in the Kalamazoo Consensus Statement (Makoul, 2001).” *Haskard 2008*
**3**	**What (materials)**	a) Materials	“Control physicians received a brochure on prostate cancer screening that was distributed by the Centers for Disease Control and Prevention, whereas intervention physicians were exposed to an interactive, 30-minute, Web-based curriculum that included interactive roulette wheels,16 illustrative video vignettes, and other content to illustrate the potential harms, benefits, and downstream consequences of receiving prostate cancer screening, as well as methods of enhancing shared decision making.” *Feng 2013*
		b) Where they can be accessed (URL, appendix)	“The clinician can obtain an estimate of the patient’s 45 day pretest probability for acute coronary syndrome and download the decision aid corresponding to the appropriate level of risk at http://shareddecisions.mayoclinic.org/decision-aid-information/chest-pain-choice-decision-aid/. Write the patient’s name in the top left corner, and give the decision aid to the patient for subsequent review.” *Hess 2016*
**4**	**What (procedures)**	Procedure and/or Activities and/or Processes	“Patients randomly assigned to Group 1 (intervention) received three, two-hour trainings in active participation, patient empowerment, and communication. The 3 trainings occurred at approximately 2 weeks, 1–2 months, and 3–4 months after enrollment. The curriculum was developed in Namibia by local content experts and was framed by the social cognitive theory of self-efficacy [references]. The content was translated into the local Namibian languages of each participating region and site. Session 1: Learning to Speak to Providers begins to teach patients how to ask questions and explain their symptoms to doctors… All trainings were held on-site, at the ART facility, in a designated clinic space that was private and large enough for groups of five to six individuals. Six months after their enrollment date, participants in the control group (Group 2) were also offered training sessions as an ethically important intervention benefit.” *Maclachlan 2016*
**5**	**Who provided**	a) Intervention provider	“Two facilitators employed by the primary care trust delivered the training and also provided access to self management support activities and resources in the primary care trust.” *Kennedy 2013*
		b) Intervention provider’s expertise and/or background	“Nurse educators were trained to adopt a neutral stance regarding the performance of prostate cancer screening.” *Myers 2011*
**6**	**How**	Mode of delivery	“Physician training was delivered in small groups and office data collection depended upon the scheduling of research assistants.” *Haskard 2008*
**7**	**Where**	a) Setting	“The patient intervention and accompanying surveys were delivered to participants prior to regularly scheduled medical appointments in a private room in each practice.” *Sheridan 2012*
		b) Location	“The trial took place at 11 primary care and family medicine sites within the Mayo Clinic Health System and Olmsted Medical Center, all in southeast Minnesota.” *Mullan 2009*
		c) Infrastructure or relevant features	“Three of the four sites provided a computer for patient use at the office but the fourth required patient access to a computer at home or elsewhere.” *Roter 2012*
**8**	**When and how much**	a) When	“Clinicians in the intervention group were to use the decision aid during the consultation with their patients, while clinicians in the control arm did not have access to the decision aid (usual care).” *LeBlanc 2015*
		b) Frequency	“PCOMS therapists received 12 h of training during two days, with four weeks apart, with respectively eight and four hours of training.”*Rise*, *2012*
		c) Duration	“The decision aid took between 11 and 34 minutes to complete, depending on which modules users chose to review.” *Schroy 2016*
**9**	**Tailoring**	If the intervention was planned to be personalised or adapted, and how.	“Building on previously developed evaluative guidelines we designed and piloted two different versions of a decision aid. Both versions included individualised risk and benefit presentation and a section to support shared decision-making.” *Thomson 2007*
**10**	**Modifications**	If the intervention was modified during the course of the study, and how.	“One version used explicit value elicitation employing the standard gamble method and a Markov decision analysis (“explicit tool”), the other included only the risk/benefit presentation (“implicit tool”). Early in the trial, the observational study showed that participants in the explicit arm found the elicitation of utilities using the standard gamble to be difficult, so this arm was discontinued (see Murtagh *et al*.) [references].” *Thomson 2007*
**11**	**Fidelity (Planned)–If assessed**	a) How and by whom intervention fidelity was assessed	“We also assessed, by reviewing the video-recorded encounters, the fidelity with which the decision aid was delivered and used as intended during these encounters using the osteoporosis fidelity checklist” *Leblanc 2015b*
		b) Strategies were used to maintain or improve fidelity	-
**12**	**Fidelity (Actual)–If assessed**	Extent to which the intervention was delivered as planned	“We also found that the fidelity with which the decision aid items were covered was high in the Decision Aid arm [67%, 95%CI (63, 78)]” *Leblanc 2015b*

To inform our qualitative observations for the latter two items, we referred to the notion of implementation fidelity as proposed by Carroll et al. [26]. Implementation fidelity refers to the degree to which an intervention or program is delivered as intended. This is important to gain a better understanding of how and why an intervention works, and the extent to which outcomes can be improved [26]. The measurement of implementation fidelity is the measurement of adherence, i.e. how far those responsible for delivering an intervention, or else its participants, actually adhere to the intervention as outlined by its designers. Adherence includes the subcategories of content, frequency, duration and coverage (also defined as “dose”) [26].

#### Procedure

Data extractors were given extraction instructions in an information session given by TTA, during which they pretested extraction methods on two studies. The extraction was performed by TTA, PR, ELA, AB, ATN, JVAF, MVRY and RA.

Pairs of extractors were chosen and each pair member extracted data from individually assigned studies. Pair members then met to compare and discuss extraction until consensus was reached. Any discrepancies in coding between pairs of coders were resolved by RA who was the designated conflict resolver.

#### Data extraction

For each study, we extracted intervention content according to the target group. In an Excel file we recorded the name of the first author, year, target group (patients, health professionals, or both) and each TIDieR item. To assess reporting of the 12 items we used a methodology developed by Tie P. Yamato and al. (2018) [27]. Each item was assessed on a 3-point scale with the following categories: not reported (NR), incompletely reported (IR), and adequately reported (R). For items classified R or IR, extractors quoted the author. For items containing sub-items (items 3, 5, 7, 8 and 11, see Table 1), all sub-items needed to be adequately reported to give the item the score “R”. If the item criterion was only achieved for one of the sub-items, the item as a whole was scored as “IR” (See S1 Table). Interventions targeting both health professionals and patients needed a unique reporting assessment so we merged their attributed categories. For instance, to declare Item 2-“Why?” adequately reported (“R”) in studies targeting both patients and health professionals, the rationale had to be adequately reported for both. The TIDieR items used for data collection and quotations representing items and sub-items from the intervention studies are presented in Table 1, which also lists the elements of each item extracted.

As not all items could be treated equally (the reporting of some items being conditional), we were not able to calculate an overall reporting score. Therefore, we present here the percentage of reporting of each item for each target group, and then of each item for the three groups as a whole. However, the results need to be interpreted carefully. “Not reported” for items 9 to 12 could represent a lack of description of the intervention (as for items 1 to 8) or that the intervention was not planned to be tailored, modified, or assessed.

The interventions were described using TIDieR by both researchers and service deliverers (clinicians, nurses etc.) to represent diverse voices [28].

### Data analysis

We performed a descriptive analysis using frequency counts (number and percentage). For each target population of the interventions (patients, health professionals, and both) we recorded levels of reporting for each of the 12 items in the TIDieR checklist. We calculated the percentage of studies in which interventions were adequately reported (R), incompletely reported (IR) or not reported (NR) in general and with respect to the target population of the interventions. Analyses were performed using version 9.4 of SAS software. For items classified R or IR, we quoted the author and performed descriptive analyses of the extracted quotes.

## Results

In the Cochrane systematic review on interventions to increase adoption of SDM by health professionals (version 2018), 87 intervention studies were included, of which 44 studies (51%) targeted only patients, 15 studies (17%) only health professionals, and 28 studies (32%) both health professionals and patients. Types of studies included were randomized trials (n = 83), non-randomized trials (n = 3) and a controlled before-after study (CBAs) (n = 1). They included intervention studies on SDM training programs as well as on SDM tools such as decision aids. See S2 Table for description of the 87 SDM interventions.

### Adequacy of reporting per item

In general, of the 87 SDM interventions the percentage of items reported was as follows: Items with most complete reporting were: “brief name” (87/87, 100%), “why” (rationale) (86/87, 99%), and “what” (procedures) (81/87, 93%). Items with least complete reporting (under 50%) were “materials” (29/87, 33%), “who” (23/87, 26%), and “when and how much” (18/87, 21%); and the conditional items “tailoring” (8/87, 9%), “modifications” (3/87, 4%), “how well (actual)”, i.e. delivered as planned? (3/87, 3%). No intervention completely reported on “how well (planned—e.g. how was fidelity improved?) (Table 2). However, we could not always tell if studies had met the condition for these items to apply. Without eliminating the conditional items, the overall completeness of reporting would have been 41.5%. If items 9 to 12 are eliminated because of their conditionality, the overall completeness of reporting was 60.2%.

**Table 2:** Level of reporting of TIDieR items in SDM interventions.

Target group (n)	Patients (44)			Health professionals (15)			Both (28)			All (N = 87) (patients, health professionals and both)		
Item number and meaning	R* n (%)	IR n (%)	NR n (%)	R n (%)	IR n (%)	NR n (%)	R n (%)	IR n (%)	NR n (%)	R n (%)	IR n (%)	NR n (%)
**1-Brief name**	44 (100)	-	-	15 (100)	-	-	28 (100)	-	-	87 (100)	-	-
**2-Why**	44 (100)	-	-	14 (93)	-	1 (7)	28 (100)	-	-	86 (99)	-	1 (1)
**3-What (materials)**	23 (52)	19 (43)	2 (5)	4 (27)	9 (60)	2 (13)	2 (7)	25 (89)	1 (4)	29 (33)	53 (61)	5 (6)
**4-What (procedures)**	44 (100)	-	-	14 (93)	1 (7)	-	23 (82)	5 (18)	-	81 (93)	6 (7)	-
**5-Who**	12 (27)	26 (59)	6 (14)	8 (53)	4 (27)	3 (20)	3 (11)	23 (82)	2 (7)	23 (26)	53 (61)	11 (13)
**6-How**	37 (84)	3 (7)	4 (9)	10 (67)	1 (7)	4 (26)	11 (39)	14 (50)	3 (11)	58 (66)	18 (21)	11 (13)
**7-Where**	38 (86)	4 (9)	2 (5)	-	15 (100)	-	-	28 (100)	-	38 (44)	47 (54)	2 (2)
**8-When and how much**	17 (39)	26 (59)	1 (2)	-	15 (100)	-	1 (4)	27 (96)	-	18 (21)	68 (78)	1 (1)
**9-Tailoring**	5 (11)	3 (7)	36 (82)	-	2 (13)	13 (87)	3 (11)	5 (18)	20 (71)	8 (9)	10 (12)	69 (79)
**10-Modifications**	3 (7)	-	41 (93)	-	-	15 (100)	-	-	28 (100)	3 (4)	-	84 (96)
**11-How well (planned)**	-	8 (18)	36 (82)	-	-	15 (100)	-	8 (29)	20 (71)	-	16 (18)	71 (82)
**12-How well (actual)**	3 (7)	25 (57)	16 (36)	-	13 (87)	2 (13)	-	21 (75)	7 (25)	3 (3)	59 (68)	25 (29)

* R = adequately reported; IR = incompletely reported; NR = not reported.

### Adequacy of reporting per target population

Overall, when comparing reporting per item per intervention target (patients, health professionals or both), interventions targeting patients were better reported than the others. For example, 84% of patient-targeted intervention studies reported “How” (delivery modes), vs. 67% for health professionals, vs. 32% for both. Only “Who,” was better reported for interventions targeting health professionals (full details in Table 2).

### Reporting levels over time

**Fig 1:**
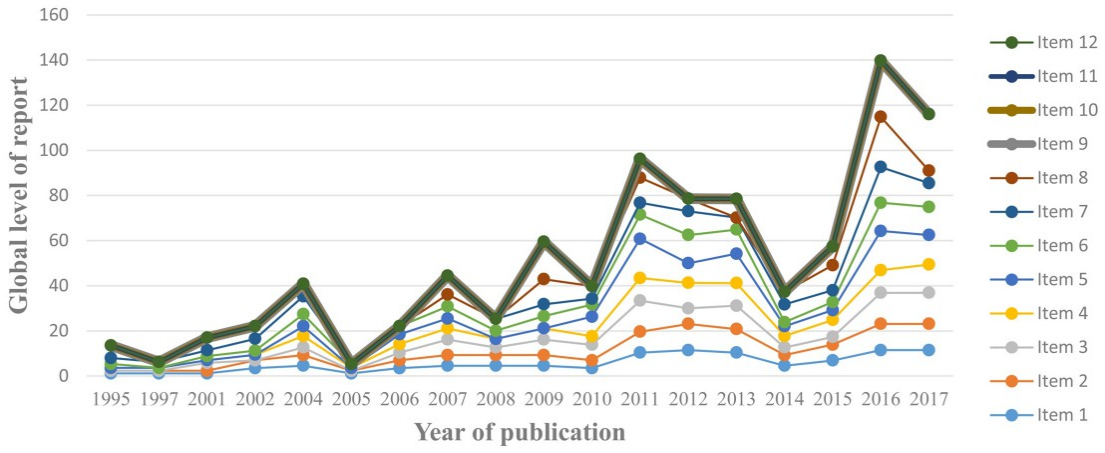
Level of reporting of TIDieR items by year of publication.

Studies were published from 1995 to 2017. In isolation, there was no clear trend in the level of reporting of each item by year of publication. But when reporting of items was compiled as a whole and compared by year, there was an upward trend in reporting levels as the years progressed (Fig 1). Since 2017 they have likely progressed further.

### Qualitative reporting per item

#### Item 3-materials

Some authors provided a URL (link) or appendix to access the materials. Four out of the 23 links (17%) provided were no longer functional [29–32]. Among the functional links, seven (30%) led to a home page [33–39] from which it was not always clear where to go for the material. For 3 others, or about 16%, access to the material on the URL provided required a username and password [40–42].

#### Item 5-who provided

Of the 44 studies on patient-targeted interventions, 26 incompletely reported this item, e.g. did not report the expertise, background or any specific training given to the people who provided the intervention. Twenty of the 26 (77%) were interventions provided by clinicians (e.g. general practitioners, surgeons, nurses, endocrinologists) [39, 42–51], researchers [32, 52–56], clinician-researchers [57, 58] or both clinicians and researchers [36]. In three of the patient-targeted interventions where interventions were mailed to participants, authors did not provide any information about the person in charge of sending emails [31, 59, 60]. For the 28 studies on interventions targeting both health professionals and patients, 23 studies incompletely reported this item. Among these, nine studies [35, 61–68] provided full information for one target group (patients) but not for the other (health professionals). In ten of the 23 studies (43%) the interventions were provided by a member of the research team or project staff (e.g. study coordinator, lead investigator) [38, 39, 69–76]. This was also the case in two out of four studies that incompletely reported this item in the 15 studies on interventions targeting health professionals [77, 78].

#### Item 6-how

Eighteen studies overall incompletely reported this item. In six of these [52, 58, 66, 70, 77, 79], authors incompletely reported how one among the multiple intervention components was delivered. Twelve of the 15 studies on interventions targeting both patients and health professionals provided complete information for either the patients [35, 38, 61–64, 68, 71, 80, 81] or the health professionals [65, 82], but not both.

#### Item 7-where

In studies on interventions targeting health professionals (including studies targeting both health professionals and patients), most authors reported the clinical setting (e.g. primary care) but not the location where the intervention occurred (e.g. consulting room). As location was not completely reported, the infrastructure or relevant features of the location was missing. For interventions targeting patients, both setting and location were reported.

#### Item 8-when and how much

Compared to the “How much”, the “When” was rarely reported for interventions targeting health professionals, whether health professionals alone (13/15 studies, or 87%) or both health professionals and patients (21 studies out of 28 studies, or 75%), whereas in patient studies, the item was completely reported at 39% or incompletely reported at 59%.

#### Item 9-tailoring

Eighteen studies overall reported a form of tailoring [30, 36, 39, 50, 51, 55–57, 66, 70, 73, 75, 82–87]. Eight tailoring studies targeted patients [30, 36, 50, 51, 55–57, 85], two targeted healthcare professionals [83, 87] and eight targeted both [39, 66, 70, 73, 75, 82, 84, 86]. While ten studies incompletely reported tailoring [36, 39, 51, 55, 66, 73, 83, 84, 86, 87], eight studies reported the complete *what*, *why*, *when* and *how* of the tailoring [30, 50, 56, 57, 70, 75, 82, 85]. All studies reported *what* was tailored and *how* tailoring was performed. However, five interventions did not report *why* the need for tailoring [36, 51, 66, 83, 86] and six did not report or incompletely reported *when* tailoring occurred [36, 39, 55, 66, 83, 84, 86].

#### Item 10-modifications

Three studies reported on modifications, all of them on patient-targeted interventions [48, 85, 88]. Two specified the complete *what*, *why*, *when*, and *how* the intervention was modified during the course of the study [85] while the third did not specify *when* the intervention was modified [88].

#### Item 11-how well (planned)

Among the 16 studies that did report on this item, none reported it completely. Fifteen did not report *by whom* fidelity was assessed [33, 38, 39, 44, 46, 51, 68, 74, 76, 82, 86, 88–91]. Ten studies reported *how* they assessed fidelity to the content [38, 44, 51, 68, 76, 82, 86, 88, 90, 91], three reported *how* they assessed fidelity to coverage [33, 46, 89], two reported *how* they assessed both fidelity to content and to coverage [39, 74]. One study reported *how* and *by whom* fidelity to content was assessed, but did not report *how* and *by whom* other types of fidelity were assessed [57]. No study reported strategies used to maintain or improve fidelity. However, we do not know if the 71 studies who did not report on this item had performed an assessment at all.

#### Item 12-how well (actual)

Among the 62 studies that did report on this item, three studies reported on it completely [51, 57, 91]. These three studies reported fidelity to content and to coverage, while the other dimensions were not applicable [51, 57, 91]. Among the 59 studies that incompletely reported this item, 48 studies reported fidelity to coverage only [29, 30, 32, 33, 35, 36, 41–43, 45–47, 49, 50, 54, 55, 63, 64, 68–73, 75, 77–81, 83, 85, 87, 89, 92–106]. Four studies reported fidelity to content only [38, 44, 82, 86]. Four studies reported fidelity to content and to coverage [39, 74, 76, 90]. One study reported fidelity to content and to frequency [88]. One study reported fidelity to duration and to coverage [53]. However, we do not know if the 25 studies that did not report on this item had performed an assessment at all.

## Discussion

In this secondary analysis of SDM intervention studies published up to 2017, we analyzed the quality of the reporting of SDM interventions using the TIDieR checklist. The three items most adequately reported were a brief name, why (rationale) and what (procedures). Other than the conditional items (9–12), the least reported items were what (materials), who, and when and how much. Interventions targeting patients were better reported than those targeting health professionals or targeting both. In multi-component studies, few authors reported on all components. Without taking into account that items 9 to 12 [item 9-Tailoring, item 10-Modifications, item 12-How well (actual), item 11-How well (planned)] were conditional, overall adequacy of reporting was 41.5%. With items 9 to 12 eliminated because of their conditionality, the overall adequacy of reporting was 60.2%. Also, we found that most links to relevant materials were not usable (e.g. no longer functional, password needed). These results lead us to make the following observations.

First, although authors made some effort to report on what materials were used (61%), this item was one of the least completely reported. For studies that did not report on how to access the material, it is possible that some of the materials are copyrighted and there may be a valid reason not to share them. We found that about 17% of URLs to the material were non-functional, and 16% of those that were functional required a username and password. This is consistent with the literature where the mortality rate of URLs found in five biomedical informatics journals was about 16.3% within the first year and reached 43.2% within five years [107]. Limited access to the materials prevents others from building on this work [108], and blocks replication and scaling up of effective strategies. Assessing the quality of the materials is also important for understanding why the intervention did or did not work as expected. Care must be taken when providing links to material that could change within a short time or that reside on restricted intranets or require user authentication [107]. An improvement in digital preservation is called for, as mentioned in the CONSORT-EHEALTH checklist [109], including defining strategies to allow permanent availability of URL references in scientific articles [107]. One strategy could be to reference URLs but include other means of locating the source if the URL expires [108]. Also, archival services (e.g. Internet Archive or Google’s cache) could be used to conserve information that has become unavailable elsewhere [107].

Second, our study shows that up to 2017, authors of most of the studies we analyzed had reported incompletely on their targeted groups or components of their multi-component SDM interventions. Reporting on interventions targeting health professionals was much less rigorous than on interventions targeting patients, perhaps because researchers are more used to following data collection protocols for patient participants than for health professional participants. Describing all the components of an intervention indicates to those wishing to replicate the intervention which elements are essential as opposed to optional or incidental [22]. However, while TIDieR captures many important aspects of SDM interventions, it may miss other more complex ones. For example, there is an apparent contradiction between fidelity (staying true to the original intervention) and tailoring it to fit new contexts. Although core components of an intervention may change over time, it may be helpful to distinguish between core components of an intervention and its modifiable components [28, 110, 111]. Indeed, complex interventions pose numerous problems for evaluators, including the practical and methodological difficulties that any successful evaluation must overcome [112]. Especially in a field where interventions are both multi-component and highly heterogenous, as shown in the Cochrane review, comparing the effect of interventions is difficult. However, if the separate components of multi-component interventions are well reported, it is more feasible. It is not possible for authors of incompletely reported multi-component intervention studies to explain the observed effect of the components of their interventions, or to compare the effect of these components, or indeed to compare them to other studies [17]. This highlights the importance of following guidance [112] or standards for reporting of all components such as the standards for reporting implementation studies (StaRI) [113].

Third, the percentage of items reported for the SDM interventions overall, using the TIDieR checklist, was less than 50%. This is congruent with other studies of the quality of reporting on behavior change interventions. A report on smoking cessation interventions found that published descriptions of behavioral support mentioned fewer than half of the behavior change techniques specified in the intervention manuals [114]. Indeed, according to a review of nearly 1,000 behaviour change outcome studies, interventions were described in detail in only 5% to 30% of experimental studies [115]. In the pharmacological field, a recent analysis found that only 11% of 262 trials of cancer chemotherapy provided complete details of the trial treatments [22]. Using the CONSORT checklist, a review over 100 herbal medicine trials (in the field of alternative medicine) found an average of 38% of the information was reported [116]. Under-reporting of interventions is thus common to several areas in the world of health research. This is not only a threat to fidelity and replicability of interventions, but also prevents the scaling up of interventions that could potentially benefit much larger populations. However, while the TIDieR checklist is in theory applicable to any clinical intervention study, some details relevant to SDM interventions in particular are not considered. In reporting on materials used with patients (e.g. decision aids or question prompts), few studies included details about how these tools were developed, e.g. if they were field tested or if they complied with the International Patient Decision Aid Standards (IPDAS) criteria [117, 118]. These details could improve the reporting of the quality of the materials. Our study highlights the need to reach a consensus on how to report this type of intervention.

Fourth, the least reported items were the optional TIDieR items. Tailoring, modifications during the course of the study and planned and actual fidelity of the intervention are rarely reported. The problem here was that we could not tell if tailoring, modification or fidelity assessments had really not taken place, or if the author omitted to present them. This was a limitation of the conceptual framework used, as it gave us a misleading picture of reporting completeness. The conditional items should be subdivided into two levels. For example, the modification question should begin “Did you modify the intervention?” followed by, at a second level, “If so, describe the modifications.” Also, all experts involved in the development of StaRI agreed that fidelity to the core components of the planned intervention and any modifications or adaptations during the course of the study should be reported first, then reported in the results section [113]. However, in the 87 studies we analyzed, modifications were reported in the methodology and in the discussion but not in the results. Placing this information in the results section would encourage reporting on the results of modifications and their impacts on the results of the study. This information is necessary to reconstruct the puzzle of the study and determine causal links between actions taken and results observed.

### Strengths and limitations

The 2017 Cochrane review on SDM interventions provided us with a rich and unexplored source of data on a topic important for all implementation science researchers and publishers. Our use of TIDieR to verify the completeness of the published information on the SDM interventions and the shortcomings it highlighted underlined the importance of using reporting guidelines such as TIDieR to improve replicability and scalability.

Our study has some limitations. First, it was a secondary analysis and data were therefore not collected primarily for the purpose of this study. Second, since 2017 there has been an increase in studies on SDM in mental health. While a few were considered in this 2017 search, most were not. Thus the current recommendations do not represent/address all conditions and patient populations. Lastly, we did not contact authors of the included studies and thus our assessment is purely based on published information.

## Conclusions

In this review, we analyzed the reporting of SDM interventions using the TIDieR checklist. The results revealed not only that many items were incompletely reported, but that few authors reported on all groups targeted or all components of their SDM interventions. These results will provide guidance for the community of SDM intervention developers on what elements need better reporting to improve the replicability of their interventions.

## Supporting information

S1 Checklist PRISMA 2009 checklist. (DOC)

S1 Table Rating of sub-items. (PDF)

S2 Table Description of shared decision making interventions. (PDF)

S1 File Database. (XLSX)

## Abbreviations

CONSORT: Consolidated Standards of Reporting Trials
CONSORT-EHEALTH: Consolidated Standards of Reporting Trials of Electronic and Mobile HEalth Applications and onLine TeleHealth
DA: Decision aid
IPDAS: International Patient Decision Aid Standards
SDM: Shared decision making
SPIRIT: Standard Protocol Items: Recommendations for Interventional Trials
StaRI: Standards for Reporting Implementation Studies
TIDieR: Template for Intervention Description and Replication

## References

[1] Charles C, Gafni A, Whelan T. Shared decision-making in the medical encounter: what does it mean? (or it takes at least two to tango). Social science & medicine. 1997;44(5):681–92. doi: 10.1016/S0277-9536(96)00221-3 9032835

[2] Makoul G, Clayman M. An integrative model of shared decision making in medical encounters. Patient education and counseling. 2006;60(3):301–12. doi: 10.1016/j.pec.2005.06.010 16051459

[3] Grad R, Légaré F, Bell NR, Dickinson JA, Singh H, Moore AE, et al. Shared decision making in preventive health care: What it is; what it is not. 2017;63(9):682–4. 28904031 PMC5597010

[4] Joosten EA, DeFuentes-Merillas L, De Weert G, Sensky T, Van Der Staak C, de Jong CA. Systematic review of the effects of shared decision-making on patient satisfaction, treatment adherence and health status. Psychotherapy and psychosomatics. 2008;77(4):219–26. doi: 10.1159/000126073 18418028

[5] Hauser K, Koerfer A, Kuhr K, Albus C, Herzig S, Matthes JJ. Outcome-relevant effects of shared decision making: a systematic review. DÄI 2015;112(40):665. doi: 10.3238/arztebl.2015.0665 26517594 PMC4640070

[6] Hamann J, Langer B, Winkler V, Busch R, Cohen R, Leucht S, et al. Shared decision making for in-patients with schizophrenia. 2006;114(4):265–73. doi: 10.1111/j.1600-0447.2006.00798.x 16968364

[7] Clayman ML, Gulbrandsen P, Morris MA. A patient in the clinic; a person in the world. Why shared decision making needs to center on the person rather than the medical encounter. JPe, counseling 2017;100(3):600–4. doi: 10.1016/j.pec.2016.10.016 27780646

[8] Stiggelbout AM, Pieterse AH, De Haes JCJM. Shared decision making: Concepts, evidence, and practice. Patient Education and Counseling. 2015;98(10):1172–9. doi: 10.1016/j.pec.2015.06.022 26215573

[9] LeBlanc A, Kenny DA, O’Connor AM, Légaré FJ. Decisional conflict in patients and their physicians: a dyadic approach to shared decision making. Mdm 2009;29(1):61–8. doi: 10.1177/0272989X08327067 19196706

[10] Légaré F, Graham ID, O’Connor AC, Aubin M, Baillargeon L, Leduc Y, et al. Prediction of health professionals’ intention to screen for decisional conflict in clinical practice. 2007;10(4):364–79. doi: 10.1111/j.1369-7625.2007.00465.x 17986073 PMC5060414

[11] Frosch DL, Légaré F, Fishbein M, Elwyn G. Adjuncts or adversaries to shared decision-making? Applying the Integrative Model of behavior to the role and design of decision support interventions in healthcare interactions. JIS 2009;4(1):73. doi: 10.1186/1748-5908-4-73 19909547 PMC2781788

[12] Couët N, Desroches S, Robitaille H, Vaillancourt H, Leblanc A, Turcotte S, et al. Assessments of the extent to which health-care providers involve patients in decision making: a systematic review of studies using the OPTION instrument. Health Expectations. 2015;18(4):542–61. doi: 10.1111/hex.12054 23451939 PMC5060794

[13] Joseph-Williams N, Elwyn G, Edwards A. Knowledge is not power for patients: a systematic review and thematic synthesis of patient-reported barriers and facilitators to shared decision making. JPe, counseling 2014;94(3):291–309. doi: 10.1016/j.pec.2013.10.031 24305642

[14] Légaré F, Thompson-Leduc P. Twelve myths about shared decision making. Patient education and counseling. 2014;96(3):281–6. doi: 10.1016/j.pec.2014.06.014 25034637

[15] Kunneman M, Engelhardt EG, Ten Hove F, Marijnen CA, Portielje JE, Smets EM, et al. Deciding about (neo-) adjuvant rectal and breast cancer treatment: missed opportunities for shared decision making. Acta Oncologica. 2016;55(2):134–9. doi: 10.3109/0284186X.2015.1068447 26237738

[16] Zisman-Ilani Y, Barnett E, Harik J, Pavlo A, O’Connell M. Expanding the concept of shared decision making for mental health: systematic search and scoping review of interventions. Mental Health Review Journal. 2017;22(3):191–213. doi: 10.1108/MHRJ-01-2017-0002

[17] Légaré F, Adekpedjou R, Stacey D, Turcotte S, Kryworuchko J, Graham ID, et al. Interventions for increasing the use of shared decision making by healthcare professionals. 2018(7). doi: 10.1002/14651858.CD006732.pub4 30025154 PMC6513543

[18] Cargo M, Harris J, Pantoja T, Booth A, Harden A, Hannes K, et al. Cochrane Qualitative and Implementation Methods Group guidance series—paper 4: methods for assessing evidence on intervention implementation. Journal of clinical epidemiology. 2018;97:59–69. doi: 10.1016/j.jclinepi.2017.11.028 . 29223325

[19] Bunger AC, Powell BJ, Robertson HA, MacDowell H, Birken SA, Shea C. Tracking implementation strategies: a description of a practical approach and early findings. Health research policy and systems. 2017;15(1):1–12. doi: 10.1186/s12961-016-0162-8 28231801 PMC5324332

[20] Minh XBN, Anh VC, Byron JP, Ha VT, Long HN, An TMD, et al. Comparing a standard and tailored approach to scaling up an evidence-based intervention for antiretroviral therapy for people who inject drugs in Vietnam: study protocol for a cluster randomized hybrid type III trial. Implementation Science. 2020;15(1):64. doi: 10.1186/s13012-020-01020-z 32771017 PMC7414564

[21] Nadeem E, Olin SS, Hill LC, Hoagwood KE, Horwitz SM. Understanding the components of quality improvement collaboratives: a systematic literature review. The Milbank Quarterly. 2013;91(2):354–94. doi: 10.1111/milq.12016 23758514 PMC3696201

[22] Hoffmann TC, Glasziou PP, Boutron I, Milne R, Perera R, Moher D, et al. Better reporting of interventions: template for intervention description and replication (TIDieR) checklist and guide. Bmj. 2014;348:g1687. doi: 10.1136/bmj.g1687 24609605

[23] Equator network. About us. https://www.equator-network.org/about-us/, on november 26, 2021. w. y.

[24] Page MJ, Moher D, Bossuyt PM, Boutron I, Hoffmann TC, Mulrow CD, et al. PRISMA 2020 explanation and elaboration: updated guidance and exemplars for reporting systematic reviews. bmj. 2021;372. doi: 10.1136/bmj.n160 33781993 PMC8005925

[25] Murad MH, Wang Z. Guidelines for reporting meta-epidemiological methodology research. BMJ Evidence-Based Medicine. 2017;22(4):139–42. doi: 10.1136/ebmed-2017-110713 28701372 PMC5537553

[26] Carroll C, Patterson M, Wood S, Booth A, Rick J, Balain S. A conceptual framework for implementation fidelity. Implementation science. 2007;2(1):40. doi: 10.1186/1748-5908-2-40 18053122 PMC2213686

[27] Yamato TP, Maher CG, Saragiotto BT, Catley MJ, Moseley AM. Rasch analysis suggested that items from the template for intervention description and replication (TIDieR) checklist can be summed to create a score. JJoce 2018;101:28–34. doi: 10.1016/j.jclinepi.2018.05.014 29793002

[28] Cotterill S, Knowles S, Martindale A-M, Elvey R, Howard S, Coupe N, et al. Getting messier with TIDieR: embracing context and complexity in intervention reporting. BMC medical research methodology. 2018;18(1):1–10. doi: 10.1186/s12874-017-0458-6 29347910 PMC5774137

[29] Krones T, Keller H, Sönnichsen A, Sadowski E-M, Baum E, Wegscheider K, et al. Absolute cardiovascular disease risk and shared decision making in primary care: a randomized controlled trial. The Annals of Family Medicine. 2008;6(3):218–27. doi: 10.1370/afm.854 18474884 PMC2384995

[30] Hess EP, Hollander JE, Schaffer JT, Kline JA, Torres CA, Diercks DB, et al. Shared decision making in patients with low risk chest pain: prospective randomized pragmatic trial. bmj. 2016;355. doi: 10.1136/bmj.i6165 27919865 PMC5152707

[31] van Peperstraten A, Nelen W, Grol R, Zielhuis G, Adang E, Stalmeier P, et al. The effect of a multifaceted empowerment strategy on decision making about the number of embryos transferred in in vitro fertilisation: randomised controlled trial. Bmj. 2010;341:c2501. doi: 10.1136/bmj.c2501 20884700 PMC2948112

[32] Köpke S, Kern S, Ziemssen T, Berghoff M, Kleiter I, Marziniak M, et al. Evidence-based patient information programme in early multiple sclerosis: a randomised controlled trial. J Neurol Neurosurg Psychiatry. 2014;85(4):411–8. doi: 10.1136/jnnp-2013-306441 24104856

[33] Krist AH, Woolf SH, Johnson RE, Kerns JW. Patient education on prostate cancer screening and involvement in decision making. The Annals of Family Medicine. 2007;5(2):112–9. doi: 10.1370/afm.623 17389534 PMC1838687

[34] Lalonde L, O’Connor AM, Duguay P, Brassard J, Drake E, Grover SA. Evaluation of a decision aid and a personal risk profile in community pharmacy for patients considering options to improve cardiovascular health: the OPTIONS pilot study. International Journal of Pharmacy Practice. 2006;14(1):51–62. doi: 10.1211/ijpp.14.1.0007

[35] Loh A, Simon D, Wills CE, Kriston L, Niebling W, Härter M. The effects of a shared decision-making intervention in primary care of depression: a cluster-randomized controlled trial. Patient education and counseling. 2007;67(3):324–32. doi: 10.1016/j.pec.2007.03.023 17509808

[36] Nannenga MR, Montori VM, Weymiller AJ, Smith SA, Christianson TJ, Bryant SC, et al. A treatment decision aid may increase patient trust in the diabetes specialist. The Statin Choice randomized trial. Health Expectations. 2009;12(1):38–44. doi: 10.1111/j.1369-7625.2008.00521.x 19250151 PMC5060475

[37] Maranda MJ, Deen D, Elshafey S, Herrera M, Gold MR. Response to a patient activation intervention among Spanish-speaking patients at a community health center in New York City. Journal of health care for the poor and underserved. 2014;25(2):591–604. doi: 10.1353/hpu.2014.0110 24858870

[38] Mullan RJ, Montori VM, Shah ND, Christianson TJ, Bryant SC, Guyatt GH, et al. The diabetes mellitus medication choice decision aid: a randomized trial. Archives of internal medicine. 2009;169(17):1560–8. doi: 10.1001/archinternmed.2009.293 19786674

[39] Branda ME, LeBlanc A, Shah ND, Tiedje K, Ruud K, Van Houten H, et al. Shared decision making for patients with type 2 diabetes: a randomized trial in primary care. BMC health services research. 2013;13(1):301. doi: 10.1186/1472-6963-13-301 23927490 PMC3751736

[40] Cox ED, Jacobsohn GC, Rajamanickam VP, Carayon P, Kelly MM, Wetterneck TB, et al. A family-centered rounds checklist, family engagement, and patient safety: a randomized trial. Pediatrics. 2017;139(5):e20161688. doi: 10.1542/peds.2016-1688 28557720 PMC5404725

[41] Stacey D, Taljaard M, Drake ER, O’Connor AM. Audit and feedback using the brief Decision Support Analysis Tool (DSAT-10) to evaluate nurse–standardized patient encounters. Patient education and counseling. 2008;73(3):519–25. doi: 10.1016/j.pec.2008.07.016 18722074

[42] Jouni H, Haddad RA, Marroush TS, Brown S-A, Kruisselbrink TM, Austin EE, et al. Shared decision-making following disclosure of coronary heart disease genetic risk: results from a randomized clinical trial. Journal of Investigative Medicine. 2017;65(3):681–8. doi: 10.1136/jim-2016-000318 27993947 PMC5325770

[43] Butow P, Devine R, Boyer M, Pendlebury S, Jackson M, Tattersall MH. Cancer consultation preparation package: changing patients but not physicians is not enough. Journal of Clinical Oncology. 2004;22(21):4401–9. doi: 10.1200/JCO.2004.66.155 15514382

[44] Montori VM, Shah ND, Pencille LJ, Branda ME, Van Houten HK, Swiglo BA, et al. Use of a decision aid to improve treatment decisions in osteoporosis: the osteoporosis choice randomized trial. The American journal of medicine. 2011;124(6):549–56. doi: 10.1016/j.amjmed.2011.01.013 21605732

[45] Murray E, Davis H, Tai SS, Coulter A, Gray A, Haines A. Randomised controlled trial of an interactive multimedia decision aid on benign prostatic hypertrophy in primary care. Bmj. 2001;323(7311):493. doi: 10.1136/bmj.323.7311.493 11532845 PMC48138

[46] Causarano N, Platt J, Baxter NN, Bagher S, Jones JM, Metcalfe KA, et al. Pre-consultation educational group intervention to improve shared decision-making for postmastectomy breast reconstruction: a pilot randomized controlled trial. Supportive Care in Cancer. 2015;23(5):1365–75. doi: 10.1007/s00520-014-2479-6 25351455

[47] Hamann J, Mendel R, Meier A, Asani F, Pausch E, Leucht S, et al. “How to speak to your psychiatrist”: shared decision-making training for inpatients with schizophrenia. Psychiatric Services. 2011;62(10):1218–21. doi: 10.1176/ps.62.10.pss6210_1218 21969650

[48] Vestala H, Frisman GH. Can participation in documentation influence experiences of involvement in care decision-making? The open nursing journal. 2013;7:66. doi: 10.2174/1874434620130516002 23802031 PMC3680981

[49] Hamann J, Parchmann A, Sassenberg N, Bronner K, Albus M, Richter A, et al. Training patients with schizophrenia to share decisions with their psychiatrists: a randomized-controlled trial. Social psychiatry and psychiatric epidemiology. 2017;52(2):175–82. doi: 10.1007/s00127-016-1327-z 28040825

[50] Korteland NM, Ahmed Y, Koolbergen DR, Brouwer M, de Heer F, Kluin J, et al. Does the use of a decision aid improve decision making in prosthetic heart valve selection? A multicenter randomized trial. Circulation: Cardiovascular Quality and Outcomes. 2017;10(2):e003178. doi: 10.1161/CIRCOUTCOMES.116.003178 28228452

[51] LeBlanc A, Wang AT, Wyatt K, Branda ME, Shah ND, Van Houten H, et al. Encounter decision aid vs. clinical decision support or usual care to support patient-centered treatment decisions in osteoporosis: the osteoporosis choice randomized trial II. PloS one. 2015;10(5). doi: 10.1371/journal.pone.0128063 26010755 PMC4444262

[52] Davison BJ, Degner LF. Empowerment of men newly diagnosed with prostate cancer. Cancer nursing. 1997;20(3):187–96. doi: 10.1097/00002820-199706000-00004 9190093

[53] Dolan JG, Frisina S. Randomized controlled trial of a patient decision aid for colorectal cancer screening. Medical Decision Making. 2002;22(2):125–39. doi: 10.1177/0272989X0202200210 11958495

[54] Almario CV, Chey WD, Khanna D, Mosadeghi S, Ahmed S, Afghani E, et al. Impact of National Institutes of Health Gastrointestinal PROMIS^®^ Measures in Clinical Practice: results of a Multicenter Controlled Trial. The American journal of gastroenterology. 2016;111(11):1546. doi: 10.1038/ajg.2016.305 27481311 PMC5097031

[55] Smallwood A, Schapira M, Fedders M, Neuner J. A pilot randomized controlled trial of a decision aid with tailored fracture risk tool delivered via a patient portal. Osteoporosis International. 2017;28(2):567–76. doi: 10.1007/s00198-016-3767-4 27647529

[56] van Tol-Geerdink JJ, Leer JWH, Wijburg CJ, van Oort IM, Vergunst H, van Lin EJ, et al. Does a decision aid for prostate cancer affect different aspects of decisional regret, assessed with new regret scales? A randomized, controlled trial. Health Expectations. 2016;19(2):459–70. doi: 10.1111/hex.12369 25940277 PMC5055275

[57] van der Krieke L, Emerencia AC, Boonstra N, Wunderink L, de Jonge P, Sytema S. A web-based tool to support shared decision making for people with a psychotic disorder: randomized controlled trial and process evaluation. Journal of medical Internet research. 2013;15(10):e216. doi: 10.2196/jmir.2851 24100091 PMC3806550

[58] Davison BJ, Degner LF. Feasibility of using a computer-assisted intervention to enhance the way women with breast cancer communicate with their physicians. Cancer nursing. 2002;25(6):417–24. doi: 10.1097/00002820-200212000-00001 . 12464832

[59] Stiggelbout AM, Molewijk AC, Otten W, Van Bockel JH, Bruijninckx CM, Van der Salm I, et al. The impact of individualized evidence-based decision support on aneurysm patients’ decision making, ideals of autonomy, and quality of life. Medical Decision Making. 2008;28(5):751–62. doi: 10.1177/0272989X08321680 18626126

[60] Street RL Jr, Voigt B, Geyer C Jr, Manning T, Swanson GP. Increasing patient involvement in choosing treatment for early breast cancer. Cancer. 1995;76(11):2275–85. 8635032 10.1002/1097-0142(19951201)76:11<2275::aid-cncr2820761115>3.0.co;2-s

[61] Leighl NB, Shepherd HL, Butow PN, Clarke SJ, McJannett M, Beale PJ, et al. Supporting treatment decision making in advanced cancer: a randomized trial of a decision aid for patients with advanced colorectal cancer considering chemotherapy. Journal of Clinical Oncology. 2011;29(15):2077–84. doi: 10.1200/JCO.2010.32.0754 . 21483008

[62] Wetzels R, Wensing M, van Weel C, Grol R. A consultation leaflet to improve an older patient’s involvement in general practice care: a randomized trial. Health Expectations. 2005;8(4):286–94. doi: 10.1111/j.1369-7625.2005.00354.x 16266416 PMC5060311

[63] Harter M, Buchholz A, Nicolai J, Reuter K, Komarahadi F, Kriston L, et al. Shared decision making and the use of decision aids. Dtsch Arztebl Int. 2015;112(40):672–9. doi: 10.3238/arztebl.2015.0672 26517595 PMC4640071

[64] Mathers N, Ng CJ, Campbell MJ, Colwell B, Brown I, Bradley A. Clinical effectiveness of a patient decision aid to improve decision quality and glycaemic control in people with diabetes making treatment choices: a cluster randomised controlled trial (PANDAs) in general practice. BMJ open. 2012;2(6):e001469. doi: 10.1136/bmjopen-2012-001469 . 23129571 PMC3532975

[65] Sheridan SL, Golin C, Bunton A, Lykes JB, Schwartz B, McCormack L, et al. Shared decision making for prostate cancer screening: the results of a combined analysis of two practice-based randomized controlled trials. BMC medical informatics and decision making. 2012;12(1):130. doi: 10.1186/1472-6947-12-130 23148458 PMC3582602

[66] Cooper LA, Roter DL, Carson KA, Bone LR, Larson SM, Miller ER, et al. A randomized trial to improve patient-centered care and hypertension control in underserved primary care patients. Journal of general internal medicine. 2011;26(11):1297–304. doi: 10.1007/s11606-011-1794-6 . 21732195 PMC3208476

[67] Deinzer A, Veelken R, Kohnen R, Schmieder RE. Is a Shared Decision–Making Approach Effective in Improving Hypertension Management? The Journal of Clinical Hypertension. 2009;11(5):266–70. doi: 10.1111/j.1751-7176.2009.00112.x . 19534034 PMC8673045

[68] Bieber C, Müller KG, Blumenstiel K, Schneider A, Richter A, Wilke S, et al. Long-term effects of a shared decision-making intervention on physician–patient interaction and outcome in fibromyalgia: A qualitative and quantitative 1 year follow-up of a randomized controlled trial. Patient education and counseling. 2006;63(3):357–66. doi: 10.1016/j.pec.2006.05.003 . 16872795

[69] Wilkes MS, Day FC, Srinivasan M, Griffin E, Tancredi DJ, Rainwater JA, et al. Pairing physician education with patient activation to improve shared decisions in prostate cancer screening: a cluster randomized controlled trial. The Annals of Family Medicine. 2013;11(4):324–34. doi: 10.1370/afm.1550 23835818 PMC3704492

[70] Fiks AG, Mayne SL, Karavite DJ, Suh A, O’Hara R, Localio AR, et al. Parent-reported outcomes of a shared decision-making portal in asthma: a practice-based RCT. Pediatrics. 2015;135(4):e965–e73. doi: 10.1542/peds.2014-3167 25755233 PMC4379463

[71] Hess EP, Knoedler MA, Shah ND, Kline JA, Breslin M, Branda ME, et al. The chest pain choice decision aid: a randomized trial. Circulation: Cardiovascular quality and outcomes. 2012;5(3):251–9. doi: 10.1161/CIRCOUTCOMES.111.964791 22496116

[72] Myers RE, Daskalakis C, Kunkel EJ, Cocroft JR, Riggio JM, Capkin M, et al. Mediated decision support in prostate cancer screening: a randomized controlled trial of decision counseling. Patient education and counseling. 2011;83(2):240–6. doi: 10.1016/j.pec.2010.06.011 20619576

[73] Cooper LA, Ghods Dinoso BK, Ford DE, Roter DL, Primm AB, Larson SM, et al. Comparative effectiveness of standard versus patient-centered collaborative care interventions for depression among african americans in primary care settings: the BRIDGE study. Health Services Research. 2013;48(1):150–74. doi: 10.1111/j.1475-6773.2012.01435.x 22716199 PMC3589960

[74] LeBlanc A, Herrin J, Williams MD, Inselman JW, Branda ME, Shah ND, et al. Shared decision making for antidepressants in primary care: a cluster randomized trial. JAMA internal medicine. 2015;175(11):1761–70. doi: 10.1001/jamainternmed.2015.5214 26414670 PMC4754973

[75] Maindal H, Carlsen A, Lauritzen T, Sandbæk A, Simmons R. Effect of a participant-driven health education programme in primary care for people with hyperglycaemia detected by screening: 3-year results from the Ready to Act randomized controlled trial (nested within the ADDITION-Denmark study). Diabetic medicine. 2014;31(8):976–86. doi: 10.1111/dme.12440 24646371

[76] Warner DO, LeBlanc A, Kadimpati S, Vickers KS, Shi Y, Montori VM. Decision aid for cigarette smokers scheduled for elective surgery. Anesthesiology: The Journal of the American Society of Anesthesiologists. 2015;123(1):18–28. doi: 10.1097/ALN.0000000000000704 25978327 PMC4626302

[77] Elwyn G, Edwards A, Hood K, Robling M, Atwell C, Russell I, et al. Achieving involvement: process outcomes from a cluster randomized trial of shared decision making skill development and use of risk communication aids in general practice. Family Practice. 2004;21(4):337–46. doi: 10.1093/fampra/cmh401 15249520

[78] Kennedy A, Bower P, Reeves D, Blakeman T, Bowen R, Chew-Graham C, et al. Implementation of self management support for long term conditions in routine primary care settings: cluster randomised controlled trial. Bmj. 2013;346:f2882. doi: 10.1136/bmj.f2882 23670660 PMC3652644

[79] Barton JL, Trupin L, Schillinger D, Evans-Young G, Imboden J, Montori VM, et al. Use of low-literacy decision aid to enhance knowledge and reduce decisional conflict among a diverse population of adults with rheumatoid arthritis: results of a pilot study. Arthritis care & research. 2016;68(7):889–98. doi: 10.1002/acr.22801 26605752 PMC4879097

[80] Rise MB, Eriksen L, Grimstad H, Steinsbekk A. The short-term effect on alliance and satisfaction of using patient feedback scales in mental health out-patient treatment. A randomised controlled trial. BMC health services research. 2012;12(1):1–12. doi: 10.1186/1472-6963-12-348 23034077 PMC3502393

[81] Wolderslund M, Kofoed P-E, Holst R, Axboe M, Ammentorp J. Digital audio recordings improve the outcomes of patient consultations: a randomised cluster trial. Patient education and counseling. 2017;100(2):242–9. doi: 10.1016/j.pec.2016.08.029 27593087

[82] Tai-Seale M, Elwyn G, Wilson CJ, Stults C, Dillon EC, Li M, et al. Enhancing shared decision making through carefully designed interventions that target patient and provider behavior. Health Affairs. 2016;35(4):605–12. doi: 10.1377/hlthaff.2015.1398 27044959

[83] Jensen BF, Gulbrandsen P, Dahl FA, Krupat E, Frankel RM, Finset A. Effectiveness of a short course in clinical communication skills for hospital doctors: results of a crossover randomized controlled trial (ISRCTN22153332). Patient education and counseling. 2011;84(2):163–9. doi: 10.1016/j.pec.2010.08.028 21050695

[84] Roter DL, Wexler R, Naragon P, Forrest B, Dees J, Almodovar A, et al. The impact of patient and physician computer mediated communication skill training on reported communication and patient satisfaction. Patient Education and Counseling. 2012;88(3):406–13. doi: 10.1016/j.pec.2012.06.020 22789149

[85] Thomson RG, Eccles MP, Steen IN, Greenaway J, Stobbart L, Murtagh MJ, et al. A patient decision aid to support shared decision-making on anti-thrombotic treatment of patients with atrial fibrillation: randomised controlled trial. BMJ Quality & Safety. 2007;16(3):216–23. doi: 10.1136/qshc.2006.018481 17545350 PMC2464985

[86] Coylewright M, Dick S, Zmolek B, Askelin J, Hawkins E, Branda M, et al. PCI choice decision aid for stable coronary artery disease: a randomized trial. Circulation: Cardiovascular Quality and Outcomes. 2016;9(6):767–76. doi: 10.1161/CIRCOUTCOMES.116.002641 27803090

[87] Sanders A, Bensing J, Essed M, Magnée T, de Wit N, Verhaak P. Does training general practitioners result in more shared decision making during consultations? Patient education and counseling. 2017;100(3):563–74. doi: 10.1016/j.pec.2016.10.002 27780647

[88] Pickett SA, Diehl SM, Steigman PJ, Prater JD, Fox A, Shipley P, et al. Consumer empowerment and self-advocacy outcomes in a randomized study of peer-led education. Community mental health journal. 2012;48(4):420–30. doi: 10.1007/s10597-012-9507-0 22460927

[89] Landrey AMD, Andrews L, Bronsert M, Denberg T. Shared decision making in prostate-specific antigen testing: the effect of a mailed patient flyer prior to an annual exam. Journal of Primary Care and Community Health 2012;4(1):67–74. doi: 10.1177/2150131912447074 23799692

[90] Epstein RM, Duberstein PR, Fenton JJ, Fiscella K, Hoerger M, Tancredi DJ, et al. Effect of a patient-centered communication intervention on oncologist-patient communication, quality of life, and health care utilization in advanced cancer: the VOICE randomized clinical trial. JAMA oncology. 2017;3(1):92–100. doi: 10.1001/jamaoncol.2016.4373 27612178 PMC5832439

[91] Eggly S, Hamel LM, Foster TS, Albrecht TL, Chapman R, Harper FW, et al. Randomized trial of a question prompt list to increase patient active participation during interactions with black patients and their oncologists. Patient education and counseling. 2017;100(5):818–26. doi: 10.1016/j.pec.2016.12.026 28073615 PMC5400698

[92] Haskard KB, Williams SL, DiMatteo MR, Rosenthal R, White MK, Goldstein MG. Physician and patient communication training in primary care: Effects on participation and satisfaction. Health Psychology. 2008;27(5):513. doi: 10.1037/0278-6133.27.5.513 18823177

[93] Feng B, Srinivasan M, Hoffman JR, Rainwater JA, Griffin E, Dragojevic M, et al. Physician communication regarding prostate cancer screening: analysis of unannounced standardized patient visits. The Annals of Family Medicine. 2013;11(4):315–23. doi: 10.1370/afm.1509 23835817 PMC3704491

[94] Deschamps MA, Taylor JG, Neubauer SL, Whiting S, Green K. Impact of pharmacist consultation versus a decision aid on decision making regarding hormone replacement therapy. International Journal of Pharmacy Practice. 2004;12(1):21–8. doi: 10.1211/0022357022999

[95] Vodermaier A, Caspari C, Koehm J, Kahlert S, Ditsch N, Untch M. Contextual factors in shared decision making: a randomised controlled trial in women with a strong suspicion of breast cancer. British journal of cancer. 2009;100(4):590–7. doi: 10.1038/sj.bjc.6604916 19209172 PMC2653746

[96] Légaré F, Guerrier M, Nadeau C, Rhéaume C, Turcotte S, Labrecque M. Impact of DECISION+ 2 on patient and physician assessment of shared decision making implementation in the context of antibiotics use for acute respiratory infections. Implementation Science. 2013;8(1):144. doi: 10.1186/1748-5908-8-144 24369771 PMC3879432

[97] Van Roosmalen M, Stalmeier P, Verhoef C, Hoekstra-Weebers J, Oosterwijk-Wakka J, Hoogerbrugge-van der Linden N, et al. Randomized trial of a shared decision-making intervention consisting of trade-offs and individualized treatment information for BRCA1/2 mutation carriers. J Clin Oncol. 2004;22(16):3293–301. doi: 10.1200/JCO.2004.05.066 15310772

[98] Maclachlan EW, Shepard-Perry MG, Ingo P, Uusiku J, Mushimba R, Simwanza R, et al. Evaluating the effectiveness of patient education and empowerment to improve patient–provider interactions in antiretroviral therapy clinics in Namibia. AIDS care. 2016;28(5):620–7. doi: 10.1080/09540121.2015.1124975 26695005 PMC4841015

[99] Bernhard J, Butow P, Aldridge J, Juraskova I, Ribi K, Brown R. Communication about standard treatment options and clinical trials: can we teach doctors new skills to improve patient outcomes? Psycho-oncology. 2012;21(12):1265–74. doi: 10.1002/pon.2044 23208837

[100] Légaré F, Labrecque M, Cauchon M, Castel J, Turcotte S, Grimshaw J. Training family physicians in shared decision-making to reduce the overuse of antibiotics in acute respiratory infections: a cluster randomized trial. Cmaj. 2012;184(13):E726–E34. doi: 10.1503/cmaj.120568 22847969 PMC3447039

[101] Shepherd HL, Barratt A, Trevena LJ, McGeechan K, Carey K, Epstein RM, et al. Three questions that patients can ask to improve the quality of information physicians give about treatment options: a cross-over trial. Patient education and counseling. 2011;84(3):379–85. doi: 10.1016/j.pec.2011.07.022 21831558

[102] Koerner M, Wirtz M, Michaelis M, Ehrhardt H, Steger A-K, Zerpies E, et al. A multicentre cluster-randomized controlled study to evaluate a train-the-trainer programme for implementing internal and external participation in medical rehabilitation. Clinical rehabilitation. 2014;28(1):20–35. doi: 10.1177/0269215513494874 23858525

[103] Tinsel I, Buchholz A, Vach W, Siegel A, Dürk T, Buchholz A, et al. Shared decision-making in antihypertensive therapy: a cluster randomised controlled trial. BMC family practice. 2013;14(1):135. doi: 10.1186/1471-2296-14-135 24024587 PMC3847233

[104] Murray MA, Stacey D, Wilson KG, O’Connor AM. Skills training to support patients considering place of end-of-life care: a randomized control trial. Journal of palliative care. 2010;26(2):112–21. 20718396

[105] O’cathain A, Walters S, Nicholl J, Thomas K, Kirkham M. Use of evidence based leaflets to promote informed choice in maternity care: randomised controlled trial in everyday practice. Bmj. 2002;324(7338):643. doi: 10.1136/bmj.324.7338.643 11895822 PMC84396

[106] Adarkwah CC, Jegan N, Heinzel-Gutenbrunner M, Kühne F, Siebert U, Popert U, et al. Time-to-event versus ten-year-absolute-risk in cardiovascular risk prevention–does it make a difference? Results from the Optimizing-Risk-Communication (OptRisk) randomized-controlled trial. BMC medical informatics and decision making. 2016;16(1):152. doi: 10.1186/s12911-016-0393-1 27899103 PMC5129612

[107] Carnevale RJ, Aronsky D. The life and death of URLs in five biomedical informatics journals. International Journal of Medical Informatics. 2007;76(4):269–73. doi: 10.1016/j.ijmedinf.2005.12.001 16458066

[108] Aronsky D, Madani S, Carnevale RJ, Duda S, Feyder MT. The prevalence and inaccessibility of Internet references in the biomedical literature at the time of publication. Journal of the American Medical Informatics Association. 2007;14(2):232–4. doi: 10.1197/jamia.M2243 17213493 PMC2213465

[109] Eysenbach Gunther, CONSORT-EHEALTH Group. CONSORT-EHEALTH: Improving and Standardizing Evaluation Reports of Web-based and Mobile Health Interventions. J Med Internet Res 2011;13(4):e126. URL: https://www.jmir.org/2011/4/e126 . 22209829 10.2196/jmir.1923 PMC3278112

[110] Charif AB, Zomahoun HTV, Massougbodji J, Khadhraoui L, Pilon MD, Boulanger E, et al. Assessing the scalability of innovations in primary care: a cross-sectional study. CMAJ open. 2020;8(4):E613. doi: 10.9778/cmajo.20200030 33011682 PMC7567510

[111] Anyon Y, Roscoe J, Bender K, Kennedy H, Dechants J, Begun S, et al. Reconciling adaptation and fidelity: implications for scaling up high quality youth programs. The journal of primary prevention. 2019;40(1):35–49. doi: 10.1007/s10935-019-00535-6 30659405

[112] Craig P, Dieppe P, Macintyre S, Michie S, Nazareth I, Petticrew M. Developing and evaluating complex interventions: the new Medical Research Council guidance. Int J Nurs Stud. 2013;50(5):587–92. doi: 10.1016/j.ijnurstu.2012.09.010 23159157

[113] Pinnock H, Epiphaniou E, Sheikh A, Griffiths C, Eldridge S, Craig P, et al. Developing standards for reporting implementation studies of complex interventions (StaRI): a systematic review and e-Delphi. Implementation Science. 2015;10(1):1–10. doi: 10.1186/s13012-015-0235-z 25888928 PMC4393562

[114] Lorencatto F, West R, Stavri Z, Michie S. How well is intervention content described in published reports of smoking cessation interventions? Jn, research t 2012;15(7):1273–82. doi: 10.1093/ntr/nts266 23262584

[115] Michie S, Fixsen D, Grimshaw JM, Eccles MP. Specifying and reporting complex behaviour change interventions: the need for a scientific method. Implement Sci. 2009 Jul 16;4:40. doi: 10.1186/1748-5908-4-40 19607700 PMC2717906

[116] Gagnier JJ, Moher D, Boon H, Beyene J, Bombardier C. Randomized controlled trials of herbal interventions underreport important details of the intervention. JJoce 2011;64(7):760–9. doi: 10.1016/j.jclinepi.2010.10.005 21208777

[117] Elwyn G, O’Connor A, Stacey D, Volk R, Edwards AG, Coulter A, et al. International Patient Decision Aids Standards (IPDAS) Collaboration. Developing a quality criteria framework for patient decision aid: online international Delphi consensus process. British Medical Journal. 2006;333(7565):417–9. doi: 10.1136/bmj.38926.629329.AE 16908462 PMC1553508

[118] Elwyn G, O’Connor A. The International patient decision aids standards (IPDAS) collaboration: The checklist, the instrument, and next steps. Shared decision making in health care—achieving evidence-based patient choice: OUP London; 2009.

