# Supplementary material for: Are shared decision making studies well enough described to be replicated? Secondary analysis of a Cochrane systematic review
Source: PLoS One. 2022 Mar 16;17(3):e0265401. doi: 10.1371/journal.pone.0265401 (PMC8926249; doi:10.1371/journal.pone.0265401)
Supplement: S1 Table — (PDF) [file pone.0265401.s002.pdf]

S1 Table. Rating of sub-items.

Sub-item$_x$ 1	Sub-item$_x$ 2	Item$_x$
R	R	R
IR	R	IR
NR	R	IR
IR	IR	IR
NR	IR	IR
NR	NR	NR

x= item number
