# Supplementary material for: Are shared decision making studies well enough described to be replicated? Secondary analysis of a Cochrane systematic review
Source: PLoS One. 2022 Mar 16;17(3):e0265401. doi: 10.1371/journal.pone.0265401 (PMC8926249; doi:10.1371/journal.pone.0265401)
Supplement: S2 Table — (PDF) [file pone.0265401.s003.pdf]

S2 Table. Description of shared decision making implementation interventions.

	Primary author	Year of publication	Country	Care setting	Context	Population	Study design	Interventions
1.	Adarkwah	2016	Germany	Primary care	Cardiovascular risk prevention	Patients (n=304)	Randomized trial	1. Single intervention: Patient mediated intervention (Computerised decision aid - TTE)<br>2. Single intervention: Patient mediated intervention (Computerised decision aid - emoticon).
2.	Almario	2016	USA	Ambulatory care, specialized care	Gastrointestinal disorders	Patients (n=371)	Non-randomized trial	1. Single intervention - Patient mediated (GI PROMIS)<br>2. Usual care
3.	Ampe	2017	Belgium	Non-ambulatory care, primary care	Advance care planning, dementia care units	Health professionals (n=90)	Non-randomized trial	1. Single intervention - Educational meeting (We DECide)<br>2. Usual care (control group)
4.	Barton	2016	USA	Ambulatory care, specialized care	Rheumatoid arthritis	Patients (n=166)	Non-randomized trial	1. Single intervention - Patient mediated intervention: (adapted guide prior to visit)<br>2. Single intervention - Patient mediated intervention: (adapted guide + decision aid during visit)<br>3. Single intervention - Patient mediated intervention: Control Group

5.	Bernhard	2011	Australia, New Zealand, Switzerland, Germany, and Austria	Specialized care; Ambulatory care	Breast cancer	Health professionals (n= 62)	Clinician randomized trial	1. Multifaceted intervention: Educational meeting, audit and feedback, distribution of educational materials<br>2. Usual care (control)
6.	Bieber	2006	Germany	Specialized care and ambulatory care	Fibromyalgia syndrome	Health professionals (n=10) Patients (n=149)	Patient randomized trial	1. Multifaceted intervention: Educational meeting with physician (18 hours); patient mediated intervention (computer-based visualized information tool)<br>2. Single intervention (control): Patient-mediated intervention (computer-based visualized information tool)
7.	Branda	2013	USA	Primary care; Ambulatory care	Type 2 diabetes	Health professionals (n= 41) Patients (n=110)	Cluster-randomized trial	1. Multifaceted intervention: Patient-mediated intervention, Educational meeting.<br>2. Usual care (control)
8.	Butow	2004	Australia	Specialized care; ambulatory care	Cancer	Patients (n=164)	Patient-randomized trial	1. Single intervention: Patient-mediated intervention (consultation preparation package:<br>2. Single intervention (control): Patient-mediated intervention (booklet NSWCancer council booklet on living with cancer)

9.	Causarano	2014	Canada	Specialized care	Post-mastectomy breast reconstruction	Patients (n=41)	Patient-randomized trial (pilot)	1. Single intervention: Patient mediated intervention (Routine education + educational meeting to patient)<br>2. Single intervention (control): Patient mediated intervention (Routine education)
10.	Cooper	2011	USA	Primary care, Ambulatory care	Hypertension	Health professionals (n=41) Patients (n=279)	Randomized trial (factorial design)	1. Patient-mediated intervention, educational meeting (Physician communication skills training and patient coaching by community health workers)<br>2. Educational meeting: Physician communication skills training<br>3. Patient-mediated intervention: patient coaching by community health workers<br>4. Patient and physician minimal intervention: (control)
11.	Cooper	2013	USA	Primary care	Major depressive disorders	Health professionals (n=36) Patients (n=132)	Provider randomized trial	1. Multifaceted intervention (Patient-centered group): Patient mediated intervention, educational outreach visit, distribution of educational material, audit and feedback<br>2. Multifaceted intervention (Standard group): Patient mediated intervention, educational outreach visit, distribution of educational material

12.	Cox	2017	USA	Non-ambulatory care, specialized care	Hospitalized children for breathing problems, gastrointestinal problems, and fever	Health professionals: Various types Patients (n=298)	Randomized trial	1. Multifaced intervention : Distribution of educational material and Educational meeting (FRC Cheklist intervention)<br>2. Usual care: page 2
13.	Coylewright	2016	USA	Ambulatory care, specialized care	Stable coronary artery disease	Health professionals (n=36) Patients (n=132)	Randomized trial	1. Single intervention - Patient mediated intervention and educational meeting<br>2. Usual care (control group)
14.	Davison	1997	Canada	Specialized care and ambulatory care (Winnipeg Community Clinic)	Prostate cancer	Patients (n=60)	Patient randomized trial	1. Single intervention; patient-mediated intervention (individual empowerment sessions)<br>2. Single intervention (control); patient-mediated intervention (information package)
15.	Davison	2002	Canada	Ambulatory care, specialized care	Oncology, breast cancer	Patients (n=749)	Randomized trial	1. Single intervention : Patient MI (computer)<br>2. Single intervention - control group : Patient mediated intervention (discussion with research nurse)
16.	Deen	2012	USA	Primary care; specialized care and ambulatory care (health center)	No one particular type of clinical condition	Patients (n=279)	Patient randomized trial	1. Patient-mediated intervention (Decision aid (DA) and Patient Activation (PA))<br>2. Patient-mediated intervention (PA)<br>3. Patient-mediated intervention (DA)<br>4. Control (doctor visit)

17.	Deinzer	2009	Germany	Specialized palliative care, non-ambulatory care	Hypertension	Healthcare professionals (n>15)<br>Patients (n=86)	Non-randomized trial	1. Multifaceted intervention: Educational meetings (training for physicians), patient mediated<br>2. Single intervention: Patient-mediated intervention (patient education program)
18.	Deschamps	2004	Canada	Primary and ambulatory care	Hormone replacement therapy	Patients (n=128)	Patient randomized trial	1. Multifaceted intervention: patient-mediated intervention (pharmacist consultation, patient-specific information and a 40-minute consultation with pharmacist) and other (a letter to the patient's physicians)<br>2. Single intervention (control): patient-mediated intervention (decision aid: "Making choices: hormones after menopause")
19.	Dolan	2002	USA	Primary and ambulatory care (two practices in Rochester New York)	Colorectal cancer screening patients	Patients (n=96)	Patient randomized trial	1. Single intervention: patient-mediated intervention (preliminary phase + detailed analysis of the decision using the analytic hierarchy process (decision aid)<br>2. Single intervention (control): patient-mediated intervention (preliminary phase and educational phase)
20.	Eggly	2017	USA	Ambulatory care, specialized care	Breast, colon, or lung cancer	Patients (n=114)	Randomized trial	1. Single intervention (QPL-only) - Patient mediated intervention

								2. Single intervention (QPL + Coach) - Patient mediated intervention<br>3. Usual Care (control group)
21.	Elwyn	2004	UK	Primary care; ambulatory care (usual practice and protected research clinics; urban and rural in Gwent, South Wales)	Non-valvular atrial fibrillation or prostatism or menorrhagia or menopausal symptoms	Healthcare professionals (n=21)<br>Patients (n=747)	Cluster-randomized trial	1. Multifaceted intervention: educational meeting (SDM skills) and audit and feedback; 5 hours<br>2. Multifaceted intervention (control): educational meeting (risk communication skills) with audit and feedback; 5 hours
22.	Epstein	2017	USA	Ambulatory care, specialized care	Non hematologic cancer	Health professionals (n=38)<br>Patients (n=265)	Randomized trial	1. Multifaced intervention Patient mediated intervention + educational meeting<br>2. Usual care
23.	Feng	2013	USA	Primary care; Ambulatory care	Prostate cancer screening	Health professionals (n=118)	Cluster-randomized trial	1. Distribution of educational material (Intervention A)<br>2. 2. Patient mediated intervention, distribution of educational material (Intervention B)<br>3. Distribution of educational material (Brochure, control)
24.	Fiks	2015	USA	Ambulatory care, primary care	Pediatric asthma	Health professionals: Various types<br>Patients (n=60)	Randomized trial	1. Multifaced intervention : Patient mediated intervention +Reminder (EHR-linked SDM portal (MyAsthma)<br>2. Single intervention - Reminder

25.	Fossli	2011	Norway	Primary care, ambulatory care	-	Healthcare professionals (n=72)	Clinician randomized trial, cross-over	1. Multifaceted intervention: educational meeting, distribution of educational materials, Audit and feedback after role-play<br>2. Usual care (Control)
26.	Hamann	2007	Germany	Specialized and non-ambulatory care (12 acute psychiatric wards of two state hospitals)	Schizophrenia	Health professionals: unknown number Patients (n=107)	Cluster-randomized trial	1. Multifaceted intervention: patient-mediated intervention (decision aid) + educational meeting with nurses, aided by various charts, lasting 30-60 minutes<br>2. Usual care (Control)
27.	Hamann	2011	Germany	Specialized and non-ambulatory care	Schizophrenia and Schizoaffective Disorder	Patients (n= 61)	Patient-randomised trial (pilot)	1. Single intervention: patient-mediated intervention (SDM training)<br>2. Single intervention (Control): patient-mediated intervention (cognitive training)
28.	Hamann	2014	Germany	Specialized and ambulatory care	Affective Disorders	Patients (n=100)	Patient-randomized trial	1. Single intervention: patient-mediated intervention<br>2. Usual care (Control): patient-mediated intervention (cognitive training)
29.	Hamann	2017	Germany	Non-ambulatory care, specialized care	Schizophrenia	Patients (n=264)	Randomized trial	1. 1. Single intervention : Patient mediated intervention (SDM Training for patients)<br>2. Single intervention : Patient mediated intervention (Training for patients)

30.	Haskard	2008	USA	Primary care; ambulatory care	Various clinical conditions	Healthcare professionals (n=156) Patients (n=2196)	Cluster-randomized trial	1. Multifaceted intervention (physician and patient trained arm): educational meeting + distribution of educational materials + patient-mediated intervention; 20 hours and 20 minutes<br>2. Multifaceted intervention (physician only trained arm): educational meeting + distribution of educational materials; 20 hours
31.	Hess	2012	USA	Tertiary care; Ambulatory care	Chest pain	Health professionals (n=102) Patients (n=204)	Patient randomized trial	1. Multifaceted intervention: patient-mediated intervention (one brief demonstration of the use of the decision aid) and educational meeting (one hour training session)<br>2. No intervention, standard care (control)
32.	Hess	2016	USA	Ambulatory care, primary care	Emergency: low risk chest pain	Patients (n=913)	Randomized trial	1. Multifaceted intervention - Patient mediated intervention (Decision aid) + reminder (quantitative pretest probability web-based tool)<br>2. Control group = usual care
33.	Härter	2015	Germany	Ambulatory and non-ambulatory care	Breast and colon cancer; male and female	Health professionals (n=86) Patients (n=160)	Provider randomized trial	1. Multifaceted intervention: patient-mediated intervention (decision aid) and educational meeting (training)<br>2. Usual care (control)

34.	Jouni	2017	USA	Ambulatory care,	coronary heart disease; male and female	Patients (n=207)	Randomized trial	1. Single intervention : Patient mediated intervention (CRS: 10-year risk of CHO based on conventional risk factors alone)<br>2. Single intervention : patient mediated intervention (CRS + GRS: conventional risk factors alone with a genetic risk score)
35.	Kasper	2008	Germany	Specialized care and ambulatory care (Hamburg University Hospital)	Multiple sclerosis	Patients (n=297)	Patient randomized trial	1. Single intervention: patient-mediated intervention (decision aid including a patient information booklet about immunotherapy options and an interactive workshop)<br>2. Single intervention (control); patient-mediated intervention (decision aid consisting of a standard information package)
36.	Kennedy	2013	United Kingdom	Primary care; Ambulatory care	Diabetes, COPD, irritable bowel syndrome	Health professionals: Various type Patients (n=5599)	Cluster-randomized trial	1. Single intervention: Educational meeting<br>2. Usual care (control)
37.	Koerner	2014	Germany	Specialized care; non-ambulatory care	Chronic diseases	Health professionals (n=363)	Cluster-randomized trial	1. Single intervention: Educational meeting<br>2. Usual care (control)

38.	Korteland	2017	Netherlands	Ambulatory care, specialized care	Prosthetic heart valve selection	Patients (n=155)	Randomized trial	1. Single intervention : Patient MI (PDA decision tool)<br>2. Usual care : standard preoperative care (control group)
39.	Krist	2007	USA	Primary care and ambulatory care (1 large family practice centre in suburban northern Virginia)	Prostate cancer screening	Patients (n=497)	Patient randomized trial	1. Single intervention: patient-mediated intervention (mailed paper version of the decision aid)<br>2. Single intervention (control): patient-mediated intervention (Internet-based decision aid)<br>3. No intervention (control)
40.	Krones	2008	Germany	Primary care; ambulatory care (CME groups in Hessen)	Cardiovascular	Health professionals (n= 91)	Clinician randomized trial	1. Multifaceted intervention: educational meeting, audit and feedback, distribution of educational materials, educational outreach visit<br>2. Single intervention (control)
41.	Köpke	2014	Germany	Ambulatory care	Clinical isolated syndrome or definite relapsing-remitting multiple sclerosis	Patients (n=192)	Patient randomized trial	1. Single intervention: Patient mediated intervention (interactive-4h education programme)<br>2. Single intervention: Patient mediated intervention (4h MS-specific stress management programme)
42.	Lalonde	2006	Canada	Primary care and ambulatory care (10 community	Cardiovascular problems	Patients (n=26)	Patient randomized trial	1. Multifaceted intervention: distribution of educational materials (decision aid + personal

				pharmacies in Montréal)				risk profile) + patient-mediated intervention (decision aid)<br>2. Multifacted intervention (control); distribution of educational materials (decision aid + personal risk assessment) + patient-mediated intervention (personal risk profile)
43.	Landrey	2012	USA	Primary care and ambulatory care	Prostate cancer screening	Patients (n=303)	Patient randomized trial	1. Single intervention (mailed flyer), patient-mediated intervention<br>2. No intervention (control)
44.	LeBlanc	2015a	USA	Primary care and ambulatory care,	Depression	Health professionals (n=117)<br>Patients (n=301)	Cluster randomized trial	1. Single intervention: patient-mediated intervention<br>2. Usual care (control)
45.	LeBlanc	2015b	USA	Ambulatory care, primary care	Osteoporosis	Health professionals (n= 41)<br>Patients (n=79)		1. Single intervention : Patient mediated intervention (Decision aid)<br>2. Single intervention : Reminder<br>3. Usual care (control)
46.	Leighl	2011	Australia, Canada	Specialized care, Ambulatory care	Advanced colorectal cancer	Health professionals (n=13)<br>Patients (n=207)	Patient randomized trial	1. Multifaceted intervention: Patient-mediated intervention (decision aid), physician training (educational meeting)<br>2. No intervention (control)

47.	Loh	2007	Germany	Primary care and ambulatory care (Department of Primary Care at University Hospital of Freiburg)	Depressive disorders	Health professionals (n=30) Patients (n=405)	Cluster randomized trial	1. Multifaceted intervention: educational meeting with physicians and patient-mediated intervention (decision aid as well as a patient information leaflet); 20 hours(educational meeting)<br>2. No intervention (control)
48.	Légaré	2012	Ambulatory care Canada	Primary care (family practise)	Acute respiratory infections	Health professionals (n=270) Patients (n=712)	Cluster randomized trial	1. Multifaceted intervention: educational meeting, distribution of educational materials (online tutorial and workshop)<br>2. Usual care (control)
49.	Maclachlan	2016	Namibia	Ambulatory care, primary care	HIV	Patients (n=592)	Randomized trial	1. Single intervention: Patient mediated intervention (Trainings for patients)<br>2. Usual care (control, wait list)
50.	Maindal	2014	Denmark	Primary care, Ambulatory care	Type 2 diabetes, impaired fasting glucose/impaired glucose tolerance	Patients (n=509)	Patient randomized trial	1. Multifaceted intervention: Patient mediated intervention, educational meeting<br>2. Single intervention (control): Educational meeting
51.	Maranda	2014	USA	Primary care, Ambulatory care	Context not specified	Patients (n=132)	Patient randomized trial	1. Single intervention: Patient mediated intervention<br>2. Usual care (control)
52.	Mathers	2012	United Kingdom	Primary care, Ambulatory care	Type 2 diabetes mellitus	Health professionals: Number: not reported;	Cluster randomized trial	1. Multifaceted intervention: Patient mediated intervention, Educational meeting<br>2. Usual care (control)

						Patients (n=175)		
53.	Montori	2011	USA	Primary care, Ambulatory care	Osteopenia/ osteoporosis	Patients (n=100)	Patient randomized trial	1. Single intervention: patient-mediated intervention; decision aid<br>2. Other single intervention (control)
54.	Mullan	2009	USA	Primary care, Ambulatory care	Diabetes type 2	Healthcare professionals (n=40)<br>Patients (n=85)	Clinician randomized trial	1. Multifaceted intervention: Patient-mediated intervention (decision aid used during the clinical encounter); and educational training (how to use decision aid)<br>2. Single intervention (control): Patient-mediated intervention (decision aid)
55.	Murray	2001	United Kingdom	Primary care and ambulatory care	Benign prostatic hypertrophy	Patients (n=112)	Patient randomized trial	1. Single-intervention: patient-mediated intervention (decision aid); 60 minutes<br>2. Usual care (control)
56.	Murray	2010	Canada	Specialized palliative care, Non-ambulatory care	Simulated patients	Healthcare professionals (n=88)	Clinician randomized trial	1. Multifaceted intervention: including educational meetings, audit and feedback, distribution of education materials; educational outreach; barriers assessment<br>2. Usual care (control)
57.	Myers	2011	USA	Primary care, Ambulatory care	Prostate cancer screening	Healthcare professionals (n=22)<br>Patients (n=313)	Patient randomized trial	1. Multifaceted intervention: Including patient-mediated interventions (pamphlet and counselling) and reminders (prompting)

								2. Multifaceted intervention: Including patient-mediated interventions and reminders (prompting) (control)
58.	Nannenga	2009	USA	Specialised care; Ambulatory care (clinic for diabetes at Mayo Clinic in Rochester, MN)	Type 2 diabetes	Patients (n=98)	Provider randomized trial (factorial 2x2 randomized trial)	1. Single intervention: decision aid administered by provider during visit<br>2. Single intervention: patient-mediated intervention (decision aid administered by researcher prior to visit)
59.	O'Cathain	2002	UK	Primary care and Ambulatory care (maternity units)	Maternity care	Health professionals: unknown number Patients (n=10,070)	Cluster-randomized trial	1. Multifaceted-intervention: education meeting with staff + distribution of educational materials ; 2 hours (educational meeting)<br>2. Usual care (control)
60.	Perestelo-Perez	2016	Spain	Ambulatory care, primary care	Diabetes	Patients (n=168)	Randomized trial	1. Single intervention: Patient mediated intervention (Decision aid)<br>2. Usual care (control)
61.	Pickett	2012	USA	Level of care: unclear; Ambulatory care	Mental illness	Patients (n=428)	Patient randomized trial	1. Single intervention: Patient mediated intervention<br>2. Usual care (control)
62.	Raynes-Greenow	2010	Australia	Specialized care (2 obstetric hospital, Sydney);	Primiparous women in their final trimester planning a vaginal birth of a	Patients (n=596)	Patient randomized trial	1. Single intervention: Patient-mediated intervention (decision aid: booklet and audio guide)

				Ambulatory care	single infant			2. Single intervention : Patient-mediated intervention (decision aid: booklet)<br>3. Single intervention (comparison group): patient-mediated (pamphlet)
63.	Rise	2012	Norway	Specialized care; Ambulatory care	Mental health	Health professionals (n=25)<br>Patients (n=75)	Patient randomized trial	1. Multifaceted intervention: Patient mediated intervention (PCOMS), Educational meeting (training of therapists)<br>2. Usual care (control)
64.	Roter	2012	USA	Primary care, Ambulatory care	Type of clinical condition not mentioned	Health professionals (n=29)<br>Patients (n=197)	Patient randomized trial	1. Multifaceted intervention: patient-mediated intervention (decision aid); distribution of educational materials<br>2. Single intervention (control): distribution of educational materials
65.	Sanders	2017	Netherlands	Ambulatory care, primary care	Back pain	Health professionals (n=42)	Randomized trial	1. Multifaceted intervention: Educational meeting + audit and feed back<br>2. Usual care (Control)
66.	Schroy	2011	USA	Primary care (Boston Medical Care centre, South Boston Community	Colorectal cancer screening	Patients (n=666)	Patient randomized trial	1. Single (first intervention group): Patient-mediated intervention (DVD audio-visual touch screen decision aid explaining screening importance, epidemiology of disease, recommended methods and their comparison, and decision

				Health Centre); Ambulatory care				guidance: Your Disease risk assessment tool with feedback)<br>2. Single intervention (second intervention group): Patient-mediated intervention (DVDaudio-visual touch screen decision aid explaining screening importance, epidemiology of disease, recommended methods and their comparison, and decision guidance)<br>3. Single intervention (control): Educational materials (a modified "9 ways to stay healthy and prevent disease")
67.	Schroy	2016	USA	Ambulatory care, primary care	Colorectal cancer	Patients (n=341)	Randomized trial	1. Multifaceted intervention : Patient mediated intervention (Decision Aid + Risk assessment tool)<br>2. Single intervention: Patient mediated intervention (Decision aid)
68.	Shepherd	2011	Australia	Primary care, Ambulatory care	Depression	Health professionals (n=36)	Randomized trial (cross-over trial)	1. Single intervention: Educational outreach visit<br>2. Usual care (control)
69.	Sheridan	2012	USA	Level of care not clear, ambulatory care	Prostate cancer screening	Health professionals (n=28)<br>Patients (n=128)	Patient randomized trial	1. Multifaceted intervention: Patient mediated intervention (video + coaching session), Educational meeting<br>2. Single intervention (control)

70.	Sheridan	2014	USA	Specialized care, Ambulatory care	Coronary heart disease risk reduction	Patients (n=160)	Patient randomized trial	1. Single intervention: Patient mediated intervention (decision aid)<br>2. Usual care (control)
71.	Smallwood	2017	USA	Ambulatory care, primary care	Osteoporosis	Patients (n=50)	Pilot randomized trial	1. Single Intervention : Patient mediated intervention (Decision aid)<br>2. Single intervention: Patient mediated intervention (Web based information)
72.	Stacey	2006	Canada	Primary care; Ambulatory care (province-wide health call centre in British Columbia)	Decisions about amniocentesis, treatment for attention deficit disorder and herniated disk, decisions about allergy injections, and treatment for gall bladder attacks and borderline hypercholesterolaemia	Health professionals (n=41) Patients: Simulated patients	Provider randomized trial	1. Multifaceted intervention: distribution of educational materials, educational meeting, as well as audit and feedback; barriers assessment; 6 hours<br>2. Usual care (control)
73.	Stiggelbout	2008	Netherlands	Specialized care and ambulatory care	Abdominal aortic aneurysm	Patients (n=113)	Patient randomized trial	1. Single-intervention: patient-mediated intervention (individualized brochure)<br>2. Single-intervention (control): patient-mediated intervention (general brochure)

74.	Street	1995	USA	Specialized care and ambulatory care (Scott and White clinic and Hospital (Texas))	Breast cancer	Patients (n=60)	Patient randomized trial	1. Single-intervention: patient-mediated intervention (Interactive multimedia program (decision aid)); 15-20 minutes<br>2. Single-intervention (control): patient-mediated intervention (brochure (decision aid))
75.	Tai-Seale	2016	USA	Primary care and ambulatory care	biomedical, health behaviour, mental health and psychosocial	Health professionals (n=26)<br>Patients (n=300)	Pilot randomized trial	1. Multifaceted intervention: Educational material (video) + Patient mediated intervention (booklet) + Educational meeting (coaching session for providers)<br>2. Multifaceted intervention: OpenCom + Patient mediated intervention (one-page ASK Handout)<br>3. Single intervention: Patient mediated intervention (one-page ASK Handout)<br>4. Usual care (Control)
76.	Thomson	2007	United Kingdom	Setting: unclear; Primary care	Atrial fibrillation	Patients (n=145)	Patient randomized trial	1. Single-intervention: Patient mediated intervention (Computerised decision aid)<br>2. Single-intervention (control): Patient mediated intervention (Guidelines)

77.	Tinsel	2013	Germany	Primary care and ambulatory care	Hypertension	Health professionals: number unknown Patients (n=1120)	Cluster randomized trial	1. Multifaceted intervention: Distribution of educational material, educational meeting<br>2. Usual care (control)
78.	van der Krieke	2013	Netherlands	Specialized and ambulatory care	Psychotic disorders	Patients (n=250)	Patient randomized trial	1. Single intervention: patient mediated intervention<br>2. Usual care (control)
79.	van Peperstraten	2010	Netherlands	Specialized care (fertilization clinics); Ambulatory care	In vitro fertilization (Client couple)	Patients: 308,	Patient randomized trial	1. Single intervention, patient-mediated intervention (decision aid, support call), reimbursement of fees; barriers assessment. Decision Aid and reimbursement; discussion; telephone call discussion<br>2. No intervention, usual care (control)
80.	van Roosmalen	2004	Netherlands	Specialized care; setting: unclear	deleterious BRCA1/2 mutation	Patients (n=88)	Cluster-randomized trial	1. Multifaceted intervention: Patient mediated intervention (SDMI+DA)<br>2. Single intervention (control): Patient mediated intervention (DA)
81.	van Tol-Geerdink	2016	Netherlands	Ambulatory care, specialized care	Prostate cancer	Patients (n=240)	Randomized trial	1. Single intervention : Patient mediated intervention (Decision aid)<br>2. Usual care (Control)
82.	Vestala	2013	Sweden	Level of care: unclear; non-ambulatory care	Chronic diseases: diabetes, inflammatory bowel	Patients (n=39)	Patient randomized trial	1. Single intervention: Patient mediated intervention<br>2. Usual care (control)

					diseases, liver disease, coronary artery disease, COPD			
83.	Vodermaier	2009	Germany	Specialized care and non-ambulatory care (gynaecological department of the University of Munich-Grosshadern;	Breast cancer	Patients (n=152)	Patient randomized trial	1. Single-intervention: Patient-mediated intervention (decision aid)<br>2. Usual care (control)
84.	Warner	2015	USA	Ambulatory and non-ambulatory care, specialized care	Preoperative context	Health professionals (n=24)<br>Patients (n=130)	Randomized trial	1. Multifaceted intervention: Patient mediated intervention (Decision Aid + Patient education brochure)+ education meeting<br>2. Single intervention: Patient mediated intervention (patient education brochure)
85.	Wetzels	2005	Netherlands	Primary care and Ambulatory care (20 practices in south-eastern Netherlands)	Various clinical conditions	Health professionals (n=25)<br>Patients (n=1246)	Cluster randomized trial	1. Multifaceted intervention: educational outreach visit, patient-mediated intervention; 30 minutes (educational outreach visit)<br>2. No intervention (control)
86.	Wilkes	2013	USA	Primary care and	Prostate cancer screening	Health professionals (n=120)	Cluster randomized trial	1. Multifaceted intervention: Patient mediated intervention, Distribution of educational material

				Ambulatory care		Patients: 712 patients + unknown number of simulated patients		2. Single intervention: Distribution of educational material<br>3. Usual care (control)
87.	Wolderslund	2017	Denmark	Ambulatory care, specialized care	Paediatrics	Health professionals (n=49)<br>Patients (n=4349)	Randomized trial	1. Multifaceted intervention: Patient mediated intervention (Question prompt list) + Other (digital audio recording)<br>2. Single intervention: Other (digital audio recording)<br>3. Usual Care

Extracted from: Légaré, F., et al., *Interventions for increasing the use of shared decision making by healthcare professionals.* Cochrane Database of Systematic Reviews, 2018(7).
